# Advances in Impedimetric Biosensors: Current Applications and Future Directions

**DOI:** 10.3390/mi16111244

**Published:** 2025-10-31

**Authors:** Ashmit Verma, Mohammad Arqam, Arwa Fraiwan

**Affiliations:** College of Engineering and Science, Louisiana Tech University, Ruston, LA 71272, USA; ave014@latech.edu (A.V.); mar107@latech.edu (M.A.)

**Keywords:** impedimetric biosensors, label-free, electrochemical detection

## Abstract

Impedimetric biosensors have emerged as a versatile class of electrochemical devices, enabling highly sensitive and real-time detection of diverse analytes. Their applications extend across healthcare diagnostics, environmental monitoring, food safety, and agriculture. By virtue of their compact size, high sensitivity, selectivity, portability, and ease of operation, these sensors have advanced rapidly in both research and practical applications. This review consolidates the wide spectrum of current applications and technological advances reported in the literature. Additionally, it examines the prospects of integrating impedimetric biosensors with emerging technology fields, including artificial intelligence, machine learning, and flexible and wearable devices. By providing an overview of the different categories of impedimetric biosensors, their detection strategies, sensing modalities, and applications, this review presents a comprehensive perspective on the current progress and future opportunities in impedimetric biosensing.

## 1. Introduction

In an era marked by global health crises, environmental pollution, and food safety concerns, the need for innovative, rapid, and reliable diagnostic technologies has never been more urgent. The modern-day challenges, such as early disease detection, pollution monitoring, and contamination-free food processing, demand solutions that are both scientifically advanced, scalable, accessible, and portable with rapid deployment and on-site analysis capabilities. Among different technological advancements and solutions, biosensors stand out as one of the most transformative analytical tools. These systems work at the interface of biology, chemistry, physics, material science, and engineering and are capable of translating complex biological interactions into measurable signals with high sensitivity, precision, and speed [1]. The term “biosensor” was first coined by Cammann, and the definition of biosensors was first introduced by the International Union of Pure and Applied Chemistry (IUPAC) [2]. With continuous advancements in the field of material science, microfabrication, and signal processing, the current biosensing modalities have redefined how we approach medical diagnostics, environmental surveillance, and industrial quality control [3].

Recently, various new modalities, such as aptamers, nanomaterials, molecularly imprinted polymers (MIPs), etc., have been introduced in the field of biosensors [4,5,6]. Additionally, advancements in the field of nanotechnology have resulted in the production of nanomaterials having a large surface-to-volume ratio that can be beneficial to biosensors in achieving higher efficiency and sensitivity [7]. As the paradigm shifts from centralized, laboratory-based analytical and testing methods to decentralized, point-of-care (PoC) and point-of-need platforms, these advanced biosensing platforms offer the potential of making the process of detection faster, more accurate, and more widely accessible than ever before [8,9].

Among various biosensing modalities, such as optical, piezoelectric, thermal, magnetic, electrical, and electrochemical. Electrochemical biosensors stand out due to their low cost, simplicity, and compatibility with miniaturized and portable systems [10,11]. Electrochemical biosensors generally consist of a three-electrode system, i.e., reference, counter electrode, and a working electrode. These biosensing systems are sophisticated analytical devices that combine biorecognition elements such as DNA, RNA, enzymes, proteins, or whole cells, with an electrochemical transduction unit to quantify and detect the analyte of interest [12]. The interaction between the biorecognition element and target analyte is highly selective, which ensures that the sensor responds only to the target substance. The primary role of the transduction unit is to convert this biochemical event, which can be a binding event or a chemical reaction, into a measurable electrical signal. Based on the nature of the signal they detect, electrochemical biosensors can be classified into three primary types: amperometric, potentiometric, and impedimetric biosensors [12]. Amperometric biosensors can detect the change in electric current resulting from redox reactions at the electrode surface. On the other hand, potentiometric biosensors can measure the changes in the electrical voltage without drawing any current. Impedimetric biosensors can monitor the changes in electrical impedance [12], and they offer key advantages over other electrochemical biosensing platforms, such as a low limit of detection (LoD), rapid measurement time, and label-free detection [13]. In contrast, amperometric biosensors, which primarily measure current, have higher LoD. Another major drawback of amperometric biosensors is their dependence on enzyme labels or redox mediators, which can increase both operational cost and assay complexity [14]. Potentiometric biosensors, while low-cost and robust, typically achieve detection limits in the micromolar (µM) to nanomolar (nM) range and are mostly limited to ion sensing rather than complex biological targets [15].

Another class of competing biosensors is plasmonic biosensors, such as those based on surface-enhanced Raman spectroscopy (SERS) or Mach-Zehnder interferometers, which can reach extraordinary sensitivities down to the single-molecule or fg/mL level for viral and protein detection, with rapid response times. Despite all the advantages, these systems generally require expensive nanostructured chips and complex optical instrumentation, which substantially increases the overall cost and system complexity [16].

Impedimetric biosensors detect subtle changes in the interfacial properties of the electrode surface, specifically resistance and capacitance induced by the detection of target analytes. These changes are typically monitored using EIS, a technique that is uniquely suited to probe both charge transfer resistance (R_ct_) and interfacial capacitance [17]. While methods like cyclic voltammetry (CV) and differential pulse voltammetry (DPV) may be employed for surface characterization or complementary analysis, they generally do not directly measure impedance [18,19]. Based on their electrochemical behavior, impedimetric biosensors can be broadly classified into two primary types, i.e., faradaic and non-faradaic systems [20]. Faradaic impedimetric biosensors involve charge transfer processes mediated by redox-active species, such as ferro/ferricyanide (Fe(CN)_6_^3−/4−^), which may be inherently present or can be introduced externally. These systems typically exhibit changes in R_ct_ upon target binding, making them highly effective for quantitative detection [21]. In contrast, non-faradaic biosensors operate without redox reactions; instead, these systems rely on changes in double-layer capacitance (C_dl_) and interfacial dielectric properties [22]. These electrochemical systems are particularly advantageous for detecting analytes in their native state, as they can avoid interference from redox agents and preserve sample integrity [23,24]. The growing interest in impedimetric biosensors is also fueled by their exceptional versatility. They can support both label-based and label-free detection strategies and can accommodate a wide variety of recognition elements, such as antibodies, aptamers, enzymes, and some other newly developed synthetic receptors like molecularly imprinted polymers (MIPs) [25,26]. Furthermore, their integration with advanced nanomaterials, microfabrication, and microfluidic technologies has significantly enhanced their sensitivity, specificity, and operability in complex or harsh environments [27,28,29].

This review aims to provide a comprehensive overview of the recent advancements in impedimetric biosensors, with a particular focus on their classification, operating mechanisms, electrochemical behaviors, recognition strategies, and applications. By evaluating the most recent advances, we highlight both the potential and limitations of this technology. We also discuss the prospects for their integration with emerging technologies such as artificial intelligence (AI), machine learning (ML), and wearable devices.

## 2. Components of Impedimetric Biosensors

Impedimetric biosensors are composed of several critical components that together define their sensitivity, selectivity, and overall performance. The choice of working electrode material [30,31,32,33], the biorecognition element [34,35,36,37,38], and the detection method all influence the biosensor’s efficiency in detecting target analytes [17,17,39]. Advances in nanomaterials, bioconjugation strategies, and electrochemical techniques have significantly enhanced the capabilities of these sensors. This section outlines the key components that enable impedimetric biosensors to function effectively in various applications.

### 2.1. Working Electrode

The working electrode in impedimetric biosensors is vital for efficient signal transduction. Gold (Au) electrodes are widely used due to their high conductivity, chemical inertness, and ease of surface modification for immobilizing biomolecules (e.g., DNA, antibodies). For example, gold interdigitated electrodes (Au-IDEs) have enabled ultra-sensitive interleukin (IL)-8 detection via non-faradaic impedance [30]. Another widely used material is Platinum (Pt). Pt electrodes provide similar conductivity to gold with superior catalytic properties, and are often used in enzymatic biosensors [31]. On the other hand, Carbon-based materials (e.g., glassy carbon, screen-printed carbon electrodes (SPCEs)), which offer the advantages of affordability and adaptability, are frequently functionalized with nanomaterials for enhanced performance [32]. Similarly, nanostructures like graphene, MXenes, and Carbon Nanotubes (CNTs) provide large surface areas and facilitate electron transfer, improving detection limits and sensitivity. For instance, graphene-based electrodes have been used for label-free impedimetric detection of cancer biomarkers, leveraging their high surface area and functionalization flexibility [33]. These advancements underline the critical role of electrode material choice in designing high-performance impedimetric biosensors. The following subsections detail the types of electrode materials, their properties, and their use in developing impedimetric biosensors.

#### 2.1.1. Metal and Metal Oxide Electrodes

Metal and metal oxide electrodes are foundational to impedimetric biosensors due to their excellent electrical conductivity, chemical stability, and compatibility with surface modification techniques for biorecognition [40].

Gold remains the most used material because of its biocompatibility and ease of functionalization via thiol-gold chemistry. For example, the recent work by Akter et al. demonstrated an ultra-sensitive impedimetric biosensor using a gold microelectrode array (Au-MEA) for early detection of cardiac troponin I (cTnI), a critical biomarker for myocardial infarction [41]. The study utilized a self-assembled monolayer of thiolated aptamers on the gold surface, achieving detection limits in the femtomolar range. The gold electrodes not only provided a stable platform for aptamer immobilization but also enabled precise impedance measurements through enhanced electron transfer. Another example is the work done by Alrebaish et al., where they developed a non-faradaic, label-free impedimetric biosensor using Au-IDEs to detect IL-8, an inflammatory biomarker [30]. Metal oxides such as indium tin oxide (ITO) or titanium dioxide (TiO_2_) are valued for their mechanical strength and transparency, enabling hybrid optical-electrochemical detection schemes. Although not the focus of Akter et al., similar metal oxide electrodes have been explored for multiplexed biosensing, where visual readouts complement impedance data [42].

#### 2.1.2. Carbon-Based Materials and Advanced Nanomaterials

Carbon-based materials, such as glassy carbon, CNTs, graphene, and graphene oxide, are widely employed in impedimetric biosensors due to their excellent electrical conductivity, high surface area, chemical stability, and ease of functionalization. These materials facilitate electron transfer processes and provide abundant binding sites for biorecognition elements, enhancing the sensitivity and selectivity of the biosensor [29,43].

For instance, Mehrban et al. report the first faradaic impedimetric sensor for the chemoprotective agent Mesna in biological samples. The sensor employs a glassy carbon electrode (GCE) modified with oxidized multi-walled carbon nanotubes (MWCNTs) and gold nanoparticles (AuNPs). In the presence of a ferricyanide redox probe, Mesna binds via its thiol group to AuNPs, causing a proportional increase in charge transfer resistance. Featuring dual linear ranges (0.06 nM–1 nM and 1 nM–130 µM) and a low detection limit of 0.02 nM, the sensor showed successful operation in blood serum and urine, demonstrating high sensitivity, selectivity, and applicability for clinical use [44].

Emerging nanomaterials such as MXenes can significantly enhance electron transfer, increase surface area, and improve signal-to-noise ratio (SNR). For example, Elnagar et al. fabricated electrospun nanofibers from sodium-alginate/polyethylene-oxide copolymers, functionalized with folic acid, on a carbon electrode. These biocompatible, antifouling nanofibers increased the electrode’s active surface area and supported specific cancer biomarker recognition. EIS results showed clear impedance shifts upon target binding, with excellent sensitivity and reproducibility. This approach illustrates how polymer nanofibers on carbon enhance both practical usability and analytical performance for impedimetric biosensing [45]. Similarly, Facure et al. developed an MXene-based “electronic tongue” electrode array on carbon substrates for simultaneous detection of multiple neurotransmitters via non-faradaic EIS. The Ti_3_C_2_T_x_ MXene layers provided high conductivity, rich surface chemistry, and rapid electron transfer. The device replicated human taste-like profiling with high sensitivity and selectivity, exemplifying carbon-MXene composites’ versatility in multiplexed, portable biosensing [46]. Likewise, Sadri et al. introduced a non-enzymatic EIS sensor on GCEs modified with 3D flower-like NiO/carbon microspheres derived from metal–organic frameworks (MOFs). The hierarchical structure increased accessible surface area (~0.093 cm^2^) and enhanced electrocatalytic activity. Impedance measurements showed linear detection of L-glutamic acid (10 µM–800 µM) with good selectivity, stability, and real-sample applicability, demonstrating a robust carbon-nanomaterial electrode without enzymes [47]. Hadian et al. fabricated a disposable carbon electrode modified with MIP monolayers confined within a Ti_3_C_2_T_x_ MXene network. This MXene-MIP architecture increased target specificity for carcinoembryonic antigen (CEA) by promoting efficient electron transfer and higher imprint fidelity. The device demonstrated high sensitivity and selectivity in a proof-of-concept EIS assay, underscoring MXene integration’s value in biomarker detection [48].

### 2.2. Biorecognition Elements

Biorecognition elements are crucial components of impedimetric biosensors, responsible for selective and specific interaction with target analytes. They define the sensor’s specificity, sensitivity, and overall analytical performance. Common biorecognition elements include: enzymes, antibodies, aptamers, DNA and RNA probes, lectins, and other biomolecules [34,35,36,37,38].

Ahmadi et al. developed a faradaic label-based impedimetric biosensor using a screen-printed gold electrode (SPGE) modified with electrodeposited AuNPs and immobilized thiol-terminated DNA to investigate DNA damage. Upon exposure to hydroxyl radicals (generated via a Fenton reaction), DNA strand breaks led to an increase in R_ct_ measured using EIS and CV. The biosensor tracked DNA damage kinetics and demonstrated the protective effect of the antioxidant deferoxamine (DFO), which reduced R_ct_ changes by scavenging radicals. This platform provides a simple, sensitive, and real-time method to monitor oxidative DNA damage and evaluate protective agents, illustrating effective use of DNA and AuNP labels in impedimetric biosensing platforms [35]. Similarly, Košelová et al. explored the enhancement of enzyme-based impedimetric biosensors by modifying electrodes with AuNPs of 20 nm and 100 nm sizes. The study evaluated how nanoparticle size and concentration affect impedance, capacitive behavior, and enzyme immobilization. The results showed that 20 nm AuNPs reduced impedance at higher glucose levels and provided a broader calibration range, while 100 nm AuNPs increased capacitive effects. The work offers insights into optimizing nanoparticle-modified biosensor design for improved detection performance [36].

Soares et al. developed a laser-induced graphene (LIG)-based label-free impedimetric immunosensor designed for rapid detection of *Salmonella* in food. The LIG electrodes, produced using laser scribing of polyimide, offer high surface area, porosity, and conductivity, enabling efficient antibody immobilization. The sensor demonstrated high sensitivity, detecting *Salmonella* in under 10 min with excellent selectivity against non-target bacteria, illustrating the potential of nanocarbon electrodes for food safety monitoring [37]. Similarly, Shoute et al. reported a portable, non-faradaic impedimetric immunosensor with Au-IDEs for detecting airborne ascospores, crucial in agricultural disease forecasting. The device achieved a LoD of ~130 aM and functioned effectively in real-time field applications [38]. The same group reported a capacitive label-free biosensor employing interdigitated (IDE) MEA for the detection of COVID-19 antibodies. The sensor enabled rapid, PoC serological testing with good reproducibility and sensitivity. This highlights the viability of micro/nanopatterned electrodes for scalable, label-free diagnostic platforms suitable for emerging infectious diseases [34].

### 2.3. Detection Methods

Detection in impedimetric biosensors is predominantly based on EIS, a powerful technique that measures the impedance of an electrode-electrolyte interface across a range of frequencies. EIS provides detailed information on the interfacial properties, such as R_ct_, C_dl_, and diffusion processes. For example, in a label-free impedimetric biosensor for prostate-specific antigen (PSA) detection, binding of PSA to immobilized antibodies on a gold electrode alters R_ct_, producing a quantifiable impedance change without the need for external labels [17]. In label-based systems, EIS can detect the amplified impedance signal caused by labels such as AuNPs or redox tags. For instance, the use of AuNP-tagged antibodies in the detection of *Helicobacter* *pylori*’s HopQ antigen enhances the impedance signal, enabling highly sensitive pathogen detection [39]. The attachment of these conductive nanoparticles alters both the R_ct_ and C_dl_, magnifying the impedance difference between bound and unbound states. CV, while not primarily an impedance technique, is often used alongside EIS to confirm the redox activity of labels or to characterize electrode surface modifications. In sensors functionalized with redox-active polymers or enzymes (e.g., glucose oxidase (GOx)), CV helps validate the electron transfer processes involved. Other techniques, such as DPV and Square Wave Voltammetry (SWV), sometimes complement EIS, especially in hybrid biosensor designs where both impedance and current responses are monitored for enhanced sensitivity. Together, these detection methods provide a versatile toolkit for probing biorecognition events, enabling the design of biosensors with high sensitivity, specificity, and adaptability for both clinical and environmental monitoring applications [17].

### 2.4. Performance Limitations and Mitigation Techniques

Impedimetric biosensors have received widespread attention for their versatility and their capacity to offer both labeled and label-free detection of a wide range of biomolecules. Despite these promising advantages, there are some performance limitations associated with these platforms that can hinder their widespread deployment in routine clinical, environmental, and industrial applications. Understanding these limitations and the strategies to tackle these challenges can be vital for advancing the field.

One of the major challenges with EIS-based biosensors is the susceptibility of the electrode surface to biofouling. As complex biological samples can have different other biological components, the adsorption of these proteins or other biomolecules irrelevant to the target analyte can block electron pathways and severely compromise sensor sensitivity and reproducibility. For example, biofouling has been shown to alter the C_dl,_ which may obscure the true binding events, resulting in decreased sensitivity and increased background noise [49]. To mitigate this problem, chemical modifications of electrode surfaces using multifunctional organic monolayer-based coatings, such as polyethylene glycol (PEG), 6-mercapto-1-hexanol (MCH), or zwitterions, were performed to minimize the nonspecific interactions. Lichtenberg et al. demonstrated that such coatings could significantly reduce protein adsorption and enhance long-term sensor stability, hence lowering LoD and improving signal-to-noise ratios [50].

Another challenge in operating impedimetric biosensors is the baseline drift. This often arises from the instability of self-assembled monolayers (SAMs), oxidation of S-Au bonds, or variations in electrode preparation, which affect the reliability of impedimetric measurements over time. A study done by Gezahagne et al. compared traditional chain alkanethiol linkers to polymer-surface linkers (o-aminobenzoic, o-ABA) on gold electrodes and found that polymer-coated electrodes demonstrated negligible drift and did not require extended stabilization periods before receptor functionalization. Additionally, they also reported that the baseline drift was found to correlate with the percentage of thiol molecules correctly bound to the electrode, emphasizing the need for rigorous electrode surface characterization and optimization in biosensor manufacturing [51].

Another limitation of impedimetric biosensors is that their sensitivity depends on uniform surface coverage and minimal roughness or defects. Bojórquez et al., in their study, observed that Au nanowire-based electrodes with heterogeneous surface properties and non-uniform roughness displayed increased impedance response variability and reduced reproducibility for SARS-CoV-2 detection. This can be overcome by optimizing fabrication techniques to achieve smoother surfaces, improved nanostructure uniformity, and higher surface coverage, which can significantly improve the performance of the biosensor [13].

Other mitigation strategies include the use of advanced nanostructured electrodes such as graphene, gold nanowire, or CNTs, to enhance electron transfer and surface area, which can boost sensitivity and lower detection limits. Moreover, the use of microfluidics has been proven to be very effective in enhancing the performance of impedimetric biosensors. Microfluidic sample pretreatment chambers are used to minimize direct exposure of electrodes to biofluids. Dong et al. combined antibody-coated magnetic nanoparticles with microfluidic cells, thereby concentrating target bacteria and improving LoD to as low as 2 cells/mL for *E. coli* O157:H7 in complex matrices [52]. The use of microfluidics also reduces the reagent consumption and analysis time while automating sample processing, essential for rapid PoC environments.

## 3. Classification of Impedimetric Biosensors

Impedimetric biosensors, owing to their versatile detection mechanisms and adaptability across a range of biorecognition schemes, can be broadly classified into two major categories: label-free and label-based systems. This classification is based primarily on the presence or absence of signal-enhancing labels such as redox tags, nanoparticles, or enzymes used during the detection process. Each type employs unique and distinct strategies to monitor impedance changes induced by the binding of the target analyte to the sensor surface.

Label-free impedimetric biosensors offer the direct measurement of the biorecognition event by measuring the inherent electrical changes at the electrode interface. On the other hand, label-based impedimetric biosensors incorporate auxiliary agents that amplify or transduce the biorecognition event into a more pronounced impedance signal.

### 3.1. Label-Free Impedimetric Biosensors

**Figure 1:**
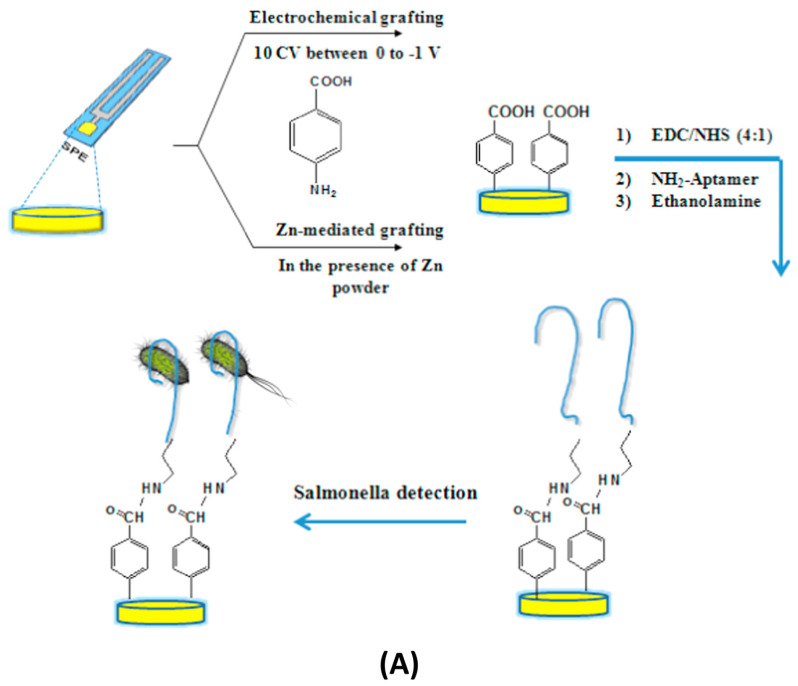

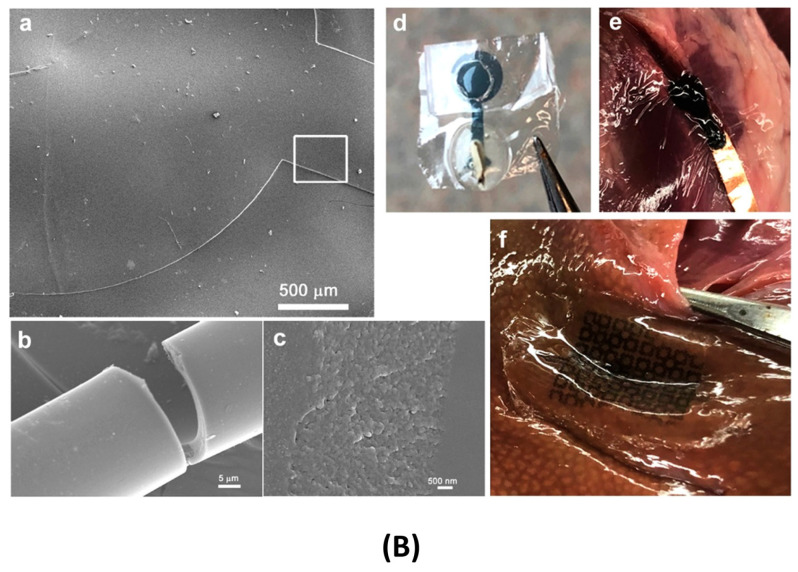
(**A**) Aptamar-based impedimetric biosensor for the detection of Salmonella (Reprinted from- Diazonium-based impedimetric aptasensor for the rapid label-free detection of *Salmonella* *Typhimurium* in food sample, Bagheryan, Z.; Raoof, J.-B.; Golabi, M.; Turner, A.P.; Beni, V. **2016**, *80*, 566–573, with permission from Elsevier) [5]. (**B**) Label-free flexible impedimetric biosensor for the detection of vascular endothelial growth factor (VEGF). (a)–(c) SEM images of the elelctrode, (d)–(f) The flexible sensor implanted in soft tissue (Reprinted with permission from [53]. Copyright 2019 American Chemical Society.

**Figure 2:**
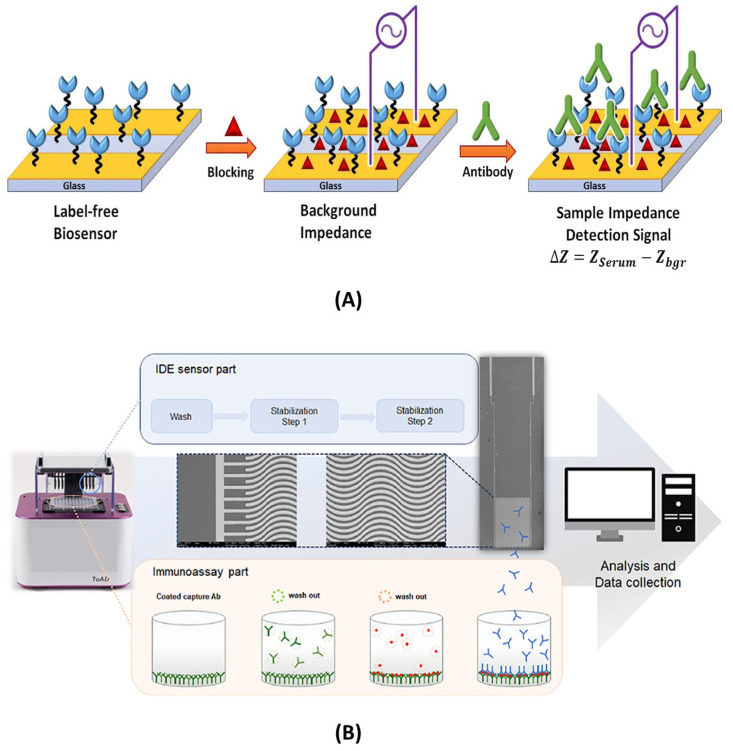
(**A**) Label-free non-faradaic impedimetric biosensor for the detection of COVID-19 antibodies (Figure is open access) [34]. (**B**) Label-free impedimetric sensor for the detection of SARS-CoV-2 nucleocapsid (N) protein (Reprinted with permission from [54] (copyright 2022 American Chemical Society).

**Figure 3:**
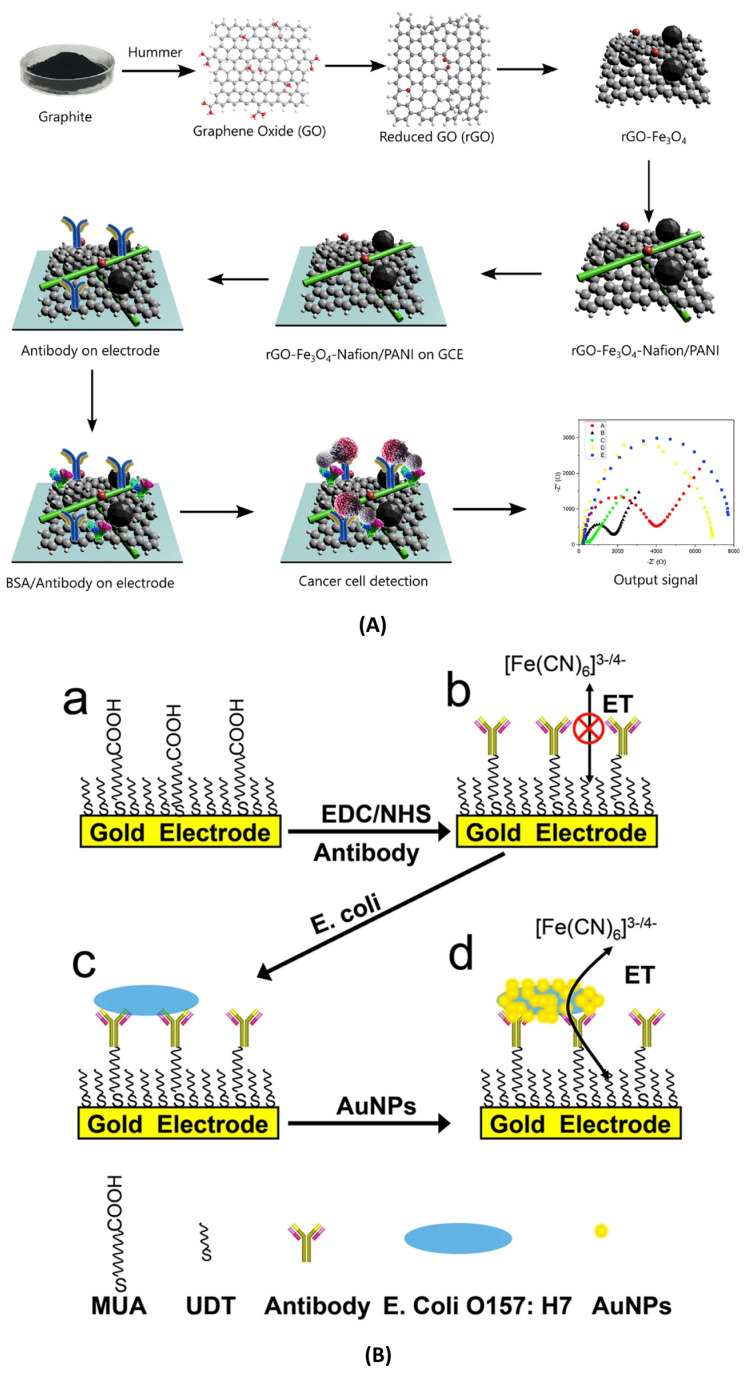
(**A**) Label-free impedimetric biosensor using a sandwich-based approach employing rGO/Fe_3_O_4_/Nafion/PANI for the detection of SK-BR3 cell line (Figure is open access) — [33] . (**B**) Label-based impedimetric biosensor for the detection of *E. coli*, (a)–(d) iluustration of the fabrication steps of the AU-NP based biosensor O157:H7 bacteria [66] (Figure is open access).

Label-free Impedimetric biosensors operate by directly monitoring alterations in the interfacial electrical properties of the sensing electrode that arise due to specific biorecognition events. These biosensors do not require any external tagging or labelling agents, consequently making the approach inherently simple, cost-effective, and well-suited for detecting analytes in their native states. Some examples of different types of label-free impedimetric biosensors are illustrated in Figure 1 , Figure 2, and Figure 3A.

The core analytical technique employed in these systems is EIS, where a small sinusoidal voltage is applied across the electrode surface over a range of frequencies, and the resulting current is measured. The interaction between the target molecule and the immobilized bioreceptor leads to changes in parameters such as R_ct_ and C_dl_, which are reflected in the impedance spectra [17].

These biosensors can be further classified based on their underlying electrochemical behavior into two primary types: faradaic (redox-active) and non-faradaic (capacitive). Faradaic systems involve electron transfer reactions between a redox-active species (e.g., ferri/ferrocyanide) in solution and the electrode. Upon analyte binding, the electron transfer kinetics are altered, resulting in a measurable change in R_ct_. This makes faradaic systems particularly effective for quantifying low-concentration targets with high precision. Fusco et al. developed a label-free impedimetric immunosensor for the detection of the herbicide 2,4-dichlorophenoxyacetic acid (2,4-D). The sensor design incorporated a nanocomposite of conductive polymer poly-(aniline-*co*-3-aminobenzoic acid) (PANABA) and MWCNTs, which were further modified with AuNPs and deposited onto IDEs. This hierarchical nanostructure provided a high surface area for antibody immobilization and improved electron transfer characteristics. Upon specific binding of 2,4-D to the immobilized antibodies, significant changes in R_ct_ were observed in the presence of a Fe(CN)_6_^3−/4−^ redox couple, confirming successful detection. The sensor achieved a low detection limit (in the ppb range), high selectivity, and good stability, highlighting the effectiveness of redox-based label-free biosensors for monitoring environmental contaminants [55].

In contrast, non-faradaic systems rely on monitoring changes in dielectric properties and interfacial capacitance at the electrode surface without using any redox reactions. The absence of redox probe minimizes the complexity of the sensing setup and preserves sample integrity, making non-faradaic systems ideal for real-time, in situ detection in complex matrices. For example, Lai et al. implemented a CMOS-based non-faradaic impedimetric biosensor to detect avian influenza H5 DNA. In their work, the IDEs were coated with dielectric thin films and modified via APTES/glutaraldehyde chemistry to immobilize DNA probes. Upon target hybridization, there was a ~13% reduction in interfacial capacitance at femtomolar concentrations, while non-complementary sequences showed negligible change [56].

These two mechanisms offer complementary advantages. For example, faradaic systems are highly sensitive and suited for quantitative assays, while non-faradaic systems provide reagent-free simplicity and strong compatibility with real-world applications.

#### 3.1.1. Recognition Elements

Label-free impedimetric biosensors utilize a diverse range of recognition elements that interact with target analytes to induce measurable changes in interfacial electrical properties. The selection of the biorecognition element is critical for determining sensitivity, selectivity, and the applications in which these biosensors can be used [4,5,6].

Aptamer-based biosensors have gained significant attention recently. This is because aptamers are short, single-stranded nucleic acids selected for high affinity and specificity toward a wide range of targets, including proteins, small molecules, and toxins. Their thermal stability, ease of synthesis, and modifiability make them well-suited for label-free detection. One notable study done by Bagheryan et al. introduced an SPCE modified with diazonium for the covalent immobilization of a *Salmonella Typhimurium*-specific aptamer. The developed biosensor demonstrated impressive analytical performance with a linear detection range of 10^1^ to 10^8^ CFU/mL and a LoD as low as 6 CFU/mL. Importantly, it was successfully tested in real samples, such as spiked apple juice, underscoring its practical applicability in food safety monitoring [5]. Another work done by Mehennaouiet al. designed a gold electrode-based aptasensor for the detection of dexamethasone (DXN), which is a corticosteroid used illegally in animal husbandry. The DXN-specific aptamer (DEX04) was immobilized on the electrode surface, and impedance changes were measured in a concentration range of 2.5–100 nM. The device achieved a LoD of 2.12 nM and exhibited excellent selectivity and reproducibility, even in milk samples, demonstrating its potential for drug residue monitoring [57]. Additionally, an advanced microfluidic-integrated aptasensor utilizing a hybrid nanomaterial-modified electrode consisting of MWCNTs, AuNPs, reduced graphene oxide (rGO) and chitosan was developed by Akbarzadeh et al. This architecture enhanced the electrode surface area and conductivity, enabling the ultrasensitive detection of oxytetracycline (OTC) with high stability, specificity, and reproducibility, making it suitable for on-site antibiotic detection in environmental and food samples [58].

MIPs are also used as the recognition element. Label-free impedimetric biosensors employing MIPs offer significant advantages in terms of robustness, reusability, and synthetic versatility. A notable example is the work done by Zhang et al., where they presented a novel label-and antibody-free impedimetric biosensor for the detection of exosomes derived from non-small cell lung cancer (NSCLC) A549 cells. The sensor capitalizes on molecular imprinting technology to create a selective recognition layer on a GCE. In their work, A549 exosomes are first immobilized onto the GCE via cholesterol anchors. Then, 3-aminophenylboronic acid (APBA) is electro-polymerized over this template. Following elution of the exosomes, the resulting MIP layer acts as a precise binding cavity for target exosomes in subsequent assays. The detection is performed using EIS, in which the exosome binding induces a measurable increase in R_ct_ of the electrode. The sensor demonstrated sensitive quantification, with a LoD of 2.03 × 10^3^ particles/mL and a limit of quantification (LoQ) of 4.1 ×10^4^ particles/mL. It showed high selectivity, distinguishing NSCLC-derived exosomes from those of healthy or other cancer cell lines and a very robust repeatability, with an average recovery ratio of ~100.76% and a resulting RSD of 1.86%. The performance of the biosensor remained stable after storage at 4 °C for one week and after seven reuse cycles [6]. This study highlights the growing relevance of MIP-based label-free impedimetric biosensors as a cost-effective and reliable alternative to biological recognition elements, particularly for small-molecule targets.

#### 3.1.2. Sensing Modalities

The sensing modality encompasses the structural and functional foundation of an impedimetric biosensor, influencing its sensitivity, response time, and integration potential with other technologies. In label-free impedimetric biosensors, the choice and design of the platform are particularly critical, as they directly affect how effectively impedance changes are captured during biorecognition events. There are several types of platforms commonly employed in this context, such as IDEs, flexible and wearable platforms, and microfluidics-integrated devices [34,54,59,60].

##### Interdigitated Electrodes

IDEs offer high surface-to-volume ratios, enhanced sensitivity, and scalable fabrication, making them ideal for developing label-free impedimetric biosensors. These platforms, especially in non-faradaic configurations, enable the real-time detection of biomarkers by capturing changes in dielectric properties without redox reactions, enhancing applicability in diagnostics, especially in miniaturized, PoC applications [59,60].

Significant progress has been made in this category recently. A work published by Shoute et al. employed an MEA to detect SARS-CoV-2 antibodies using non-faradaic impedance. In this work, the functionalization with SARS-CoV-2 spike proteins allowed specific binding to target antibodies, which altered system capacitance. The developed sensor achieved a LoD of 0.4 BAU/mL, comparable to validated commercial assays. The design was label-free, fast (under 1 h), and could enhance sensitivity by adjusting ionic strength, making it well-suited for rapid, PoC serological testing during pandemics or large-scale screenings [34]. Similar work was done by Cho et al., where a high-throughput, label-free detection method was employed for SARS-CoV-2 nucleocapsid (N) protein using an enzyme-linked immunosorbent assay (ELISA) format and 96-interdigitated electrode array (ToAD). The system quantified residual detection antibodies post-sandwich assay, which showed high specificity and minimal cross-reactivity. A LoD of 0.1 ng/mL was achieved with short turnaround time and the ability to test for multiple analytes simultaneously. The platform’s compatibility with conventional 96-well plates makes it very attractive for scalable diagnostics and adaptable to a broad range of antigen/antibody-based biosensing applications [54]. An example of a non-faradaic biosensor using IDEs was developed by Assaifan et al., for real-time detection of LDL cholesterol. In this work, the antibodies were immobilized on the surface for selective capture. The sensor demonstrated a LoD of 120 pg/mL and a sensitivity of 70 nF/log(ng/mL), making it effective for clinical use. Uniquely, it enabled continuous monitoring in PBS without a reference electrode, positioning it as a scalable, cost-effective solution for wearable biosensing and early intervention in cardiovascular disease management [59]. Recently, Alrebaish et al. developed an Au-IDE-based non-faradaic biosensor for label-free detection of IL-8, which is a key inflammation biomarker. In this work, researchers investigated several impedance parameters, including impedance magnitude (Z_mod_), real impedance (Z_real_), and imaginary impedance (Z_imag_), to determine the most sensitive metric. Z_imag_ demonstrated the best performance with a LoD of 90 pg/mL and sensitivity of 13.1 kΩ/log(ng/mL), outperforming conventional capacitance-based approaches. This work highlights the advantage of using alternative impedimetric parameters for optimizing biosensor performance and provides a platform for sensitive cytokine detection without labels [30].

##### Flexible and Wearable Platforms

Flexible and wearable impedimetric biosensors enable non-invasive, continuous monitoring of biomarkers directly from bodily fluids such as sweat or serum. These platforms integrate soft materials, stretchable electronics, and label-free electrochemical interfaces to offer real-time diagnostics suitable for personalized healthcare, remote monitoring, and early detection of physiological changes in a convenient and comfortable format [53,60,61].

For example, Lee et al. developed a wearable, stretchable lab-on-a-patch (LoP) platform for real-time, label-free detection of cortisol in sweat. It combines a 3D nanostructured gold electrode for high sensitivity with a stretchable microfluidic module for precise sweat collection and reagent delivery. The system is reusable and supports one-touch operation. It accurately detected cortisol levels during exercise, demonstrating practical on-body immunodetection. This innovative integration showcases the promise of using conformable biosensors for real-time stress and hormone monitoring in wearable healthcare tools [60]. Another work reported by Xu et al. presented a fully organic, label-free impedimetric biosensor on a flexible, biodegradable fibroin substrate to detect vascular endothelial growth factor (VEGF). Using a photoreactive silk-sericin-conducting polymer ink, patterned electrodes were created via photolithography. VEGF antibodies were immobilized within the conductive matrix, enabling specific and sensitive VEGF detection in human serum. The biosensor demonstrated excellent biocompatibility and environmental degradability. Its performance in complex biological fluids highlights its potential in wound monitoring, early disease diagnostics, and sustainable healthcare applications requiring flexible, single-use biosensors [53].

##### Microfluidic Integration

Microfluidic-integrated impedimetric biosensors combine precise fluid handling with sensitive electrochemical detection, enabling the development of miniaturized and efficient lab-on-chip platforms. These systems offer advantages such as low reagent consumption, enhanced SNR, and real-time detection, making them ideal for PoC-diagnostics, pathogen monitoring, and field-deployable sensing applications [62,63].

In the work reported by Farooq et al., they fabricated a flexible impedimetric microfluidic cytometer based on the Coulter principle to detect and differentiate cells using coplanar microelectrodes. Comparing two microfluidic geometries, the study highlighted how microelectrode positioning and sensing volume impact pulse amplitude and signal clarity. A maximum SNR of 38.78 dB enabled effective detection of 10 µm beads and leukemia cells. Additionally, a robust bonding technique between polypropylene and Polydimethylsiloxane (PDMS) layers enhanced structural reliability, supporting portable biosensor applications in medical diagnostics [61]. Another innovative work on microfluidic biosensors was done by Zhang et al. for the real-time detection of plant pathogen zoospores. Utilizing chemotaxis, *Phytophthora cactorum* spores were guided into a detection channel with attractant gradients and detected via transient impedance signals. The system was able to achieve a single zoospore resolution, with SNRs of ~17 (flow-based) and ~5.9 (gradient-induced). This label-free platform offers selective, sensitive detection in field conditions, and its low-cost, remotely operable design holds promise for use in early pathogen monitoring in agricultural applications [62].

### 3.2. Label-Based Impedimetric Biosensors

Label-based impedimetric biosensors rely on external labelling agents, such as enzymes, nanoparticles (e.g., AuNPs), redox tags, or quantum dots, to amplify the impedance response following a biorecognition event [25]. These labels either directly participate in redox reactions or influence the dielectric environment at the electrode interface, which results in measurable changes in R_ct_, capacitance, or impedance magnitude. This signal amplification results in enhanced sensitivity, particularly in complex biological samples where label-free detection may not be sufficient. The methods used for labelling typically involve covalently or non-covalently attaching the label to recognition elements (e.g., antibodies, aptamers) that selectively bind with the target analyte. The binding event either brings the label into proximity with the electrode or alters the interfacial properties in a manner that modulates the impedance signature. This enables detection at lower concentrations, with improved selectivity and robustness compared to label-free formats [64].

**Figure 4:**
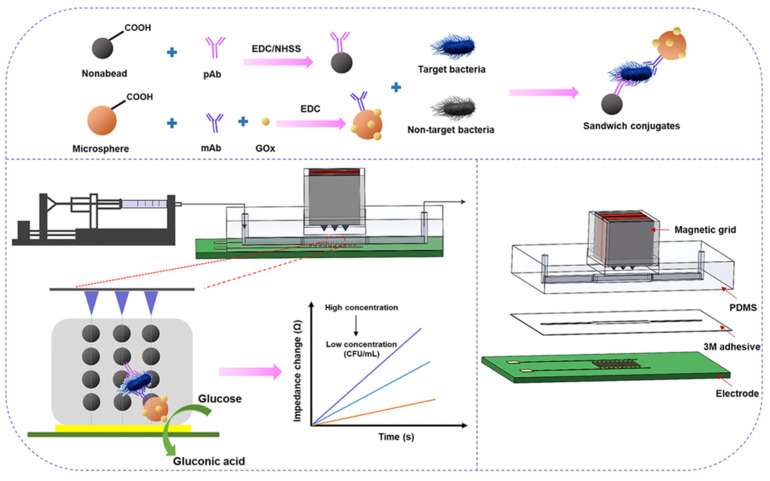
Label-based impedimetric biosensor using IDEs for the detection of *Salmonella.* (Reprinted from—Magnetic nanobead chain-assisted real-time impedance monitoring using PCB interdigitated electrode for *Salmonella* detection, Jiang, F. et al., **2023**, *26*, 108245, with permission from Elsevier) [67].

As in label-free impedimetric biosensors, label-based impedimetric biosensors can also be further classified into two primary types: faradaic and non-faradaic biosensors. The faradaic system relies on labelling agents, such as enzymes, redox tags, or nanoparticles, to introduce or generate redox-active species that amplify electron transfer at the electrode interface during biorecognition events. This enhances sensitivity and enables precise detection of target analytes [65]. Hong et al. developed an electrochemical lectin-based biosensor array designed for rapid and multiplexed detection of bacterial pathogens. In this work, an MEA was functionalized with different lectins that selectively bind to characteristic glycans on bacterial surfaces. Upon binding, faradaic impedance spectroscopy in the presence of a redox probe (ferricyanide/ferrocyanide) measured changes in R_ct_ associated with each lectin-bacteria interaction. The platform successfully differentiated Gram-positive (*Staphylococcus aureus*) and Gram-negative (*Escherichia coli*) bacteria with high specificity and sensitivity. This approach demonstrates the potential of faradaic label-based systems for parallel, label-enhanced pathogen detection in clinical diagnostics and environmental monitoring applications [63]. Examples of label-based impedimetric biosensors are illustrated in Figure 3B and Figure 4.

#### 3.2.1. Recognition Elements

Recognition elements are integral to the selectivity and sensitivity of label-based impedimetric biosensors. They provide the molecular specificity necessary for identifying target analytes in complex biological samples. Commonly employed recognition elements include antibodies, enzymes, nucleic acids, and synthetic receptors, often conjugated with signal-amplifying labels such as redox tags or nanoparticles. These components enable precise biorecognition events that translate into measurable impedance changes, facilitating applications in clinical diagnostics, environmental monitoring, and food safety with enhanced detection limits.

Antibody-based recognition elements form the foundation of many label-based impedimetric biosensors due to their high specificity and affinity toward target antigens. These immunosensors typically utilize antibodies, which are Y-shaped glycoproteins produced by the immune system to selectively bind to analytes such as proteins, pathogens, or toxins. In label-based impedimetric systems, antibodies are typically conjugated with redox tags, enzymes, or nanoparticles that generate or amplify electrochemical signals upon antigen binding. The biorecognition event induces measurable changes in impedance parameters, often enabling highly sensitive detection even in complex biological matrices. Antibody-based label strategies offer the added advantages of robustness, reproducibility, and compatibility with miniaturized biosensing platforms. One such platform was developed by Jingzhuan Wan et al., where they fabricated a faradaic impedance immunosensor which utilizes AuNP tags as electron transfer mediators (Figure 3B). In this sandwich-type format, anti-*E. coli* antibodies immobilized on a self-assembled monolayer (SAM) on gold electrodes capture *E. coli* O157:H7. A secondary antibody labelled with AuNPs binds the captured bacteria, creating electron-transfer pathways across the insulating SAM. This results in a pronounced decrease in R_ct_ in the presence of a ferro/ferricyanide redox probe. The use of AuNP labelling significantly enhanced sensitivity and produced a reliable signal-off response. This strategy showcased a labelled, faradaic immunosensor that achieves signal amplification via nanoparticle-facilitated electron transfer [66].

Similarly, enzyme-based impedimetric biosensors, which use enzymes as biorecognition elements, capitalizing on their catalytic activity to amplify impedance signals. These sensors typically involve the enzyme catalyzing a reaction that produces or consumes electroactive species, thus influencing the R_ct_ or capacitance at the electrode interface. When paired with redox mediators or labels, enzyme-based biosensors can achieve highly sensitive and specific detection of small molecules, metabolites, or disease biomarkers. This approach is well established in glucose sensing and is expanding into broader clinical and environmental applications. For example, Asrami et al. engineered a faradaic enzyme-based impedimetric biosensor by immobilizing GOx on a copper oxide (CuO)-chitosan nanocomposite coated over a fluorine-doped tin oxide (FTO) electrode. The CuO-chitosan nanostructure provides a high surface area scaffold that enhances enzyme loading and electron transfer. In the presence of a ferro/ferricyanide redox couple, glucose oxidation by GOx alters the local concentration of redox-active species, causing measurable changes in R_ct_. The sensor demonstrated a detection sensitivity of 0.261 kΩ/mM, a linear range from 0.2 to 15 mM, and a low LoD of 27 μM. Its performance indicates a strong potential for rapid, labelled, enzyme-amplified glucose monitoring in clinical diagnostics [68].

#### 3.2.2. Sensing Modalities

Typically, nanostructured electrodes and microfluidic-integrated systems are employed in label-based impedimetric biosensors to enhance the surface area, facilitate rapid mass transport, and support complex assay formats.

##### Nanostructured Electrodes with Labels

In the work done by Wang et al., a nanobiocomposite comprising CuO nanoparticles embedded within a chitosan matrix was deposited on an FTO electrode. GOx was immobilized on this platform, with ferro/ferricyanide redox probes enabling faradaic impedance detection. The nanostructured matrix provided a biocompatible environment and promoted efficient electron transfer, leading to enhanced sensitivity and stability for glucose sensing [69]. Another example is the biosensor reported by Singh et al., which utilized Prussian Blue nanoparticles incorporated into a Langmuir–Blodgett film on a gold electrode to immobilize GOx. The Prussian Blue acted as an internal redox label and mediator, amplifying the impedance signal during glucose detection. This nanostructured, label-integrated electrode demonstrated significant improvements in LoD and stability compared to conventional systems, underscoring the synergistic benefits of combining nanomaterials and labels in impedimetric biosensor design [41].

##### Microfluidic Systems

A noteworthy example of a label-based microfluidic impedimetric biosensor is the system developed by Jiang et al. for rapid detection of *Salmonella Typhimurium* using magnetic nanobead chains conjugated with alkaline phosphatase (ALP) as labels. In this design, a microfluidic channel is integrated with microelectrodes to enable real-time impedance monitoring. Functionalized magnetic nanobeads capture target bacteria, and the application of a magnetic field forms bead chains that traverse the microchannel. The ALP labels catalyze a substrate to generate electroactive products, leading to measurable Faradaic impedance changes, specifically a modulation in R_ct_. This configuration allows sensitive, specific, and rapid (~3 min) detection of *S. Typhimurium* in complex biological samples. The microfluidic setup not only facilitates precise sample handling but also supports dynamic impedance monitoring [67].

The classification, features and main performance parameters of the biosensors reviewed in this section are summarized Table 1.

## 4. Current Applications of Impedimetric Biosensors

### 4.1. Medical and Clinical Diagnostics with PoC Devices for Rapid Testing

Impedimetric biosensors have been considered a powerful, advanced, and effective tool for detecting cancer biomarkers, cardiac markers, and diabetes due to the combination of versatility, sensitivity, and practical applicability.

#### 4.1.1. Cancer Biomarker Detection

Impedimetric biosensors operate by binding a cancer biomarker to an immobilized antibody or aptamer. For cancer biomarker detection, the primary advantage of impedimetric biosensors is high sensitivity, rapid response, label-free detection and the possibility for miniaturization and integration into PoC devices, which makes them particularly reliable and highly suitable for early cancer detection [17,70,71]. Clinically relevant biomarkers, including nucleic acids, proteins and even whole cancer cells, can be detected with the use of impedimetric biosensors. For instance, immunosensors that utilize nanostructures like CNTs, AuNPs and their composites to modify surface electrodes have demonstrated high sensitivity for the detection of protein biomarkers like IL-6, PSA, and CEA. The sensors showcased the detection limits reaching as low as the picogram or femtomolar range in human serum [72]. The sensitivity of the biosensors improves significantly by integrating nanomaterials as it increases the surface area for bioreceptor immobilization and enhances electron transfer kinetics [17]. Impedimetric biosensors have also been successfully proven effective for measuring anticancer drug levels such as Mesna, in biological fluids. Mehrban et al. fabricated an impedimetric sensor in which GCEs were modified with MWCNTs and AuNPs and achieved detection limits as low as 0.02 nM, demonstrating their application in anticancer drug monitoring [44]. Recent advancements also combine impedimetric biosensors with microfluidic platforms that provide high-throughput and single-cell analysis. These integrated systems enable the capture and analysis of single cancer cells, supporting applications in drug efficacy testing and early diagnosis [73].

#### 4.1.2. Diabetes Biomarkers Detection

Impedimetric biosensors’ primary appeal lies in highly sensitive, label-free, and real-time detection of key diabetes biomarkers, such as L-tyrosine and glucose. Impedimetric glucose biosensors are one of the most extensively researched and commercially viable biosensing platforms. Biosensors being used for diabetes monitoring typically employ enzyme-based recognition, predominantly utilizing GOx confined on electrodes to catalyze the oxidation of glucose. Naderi Asrami et al. developed a biosensor based on a nanostructured CuO-chitosan composite in which an FTO substrate was used. The sensor displayed high sensitivity (0.261 kΩ/mM), a broad linear range (0.2–15 mM) and a low LoD (27 μM), thus enabling accurate glucose monitoring. The device also exhibited steady robustness over several weeks and rapid response (less than 4 s), hence confirming its practical applicability for diabetes detection in biological samples such as human serum [68]. As diabetes is multifactorial in nature, the researchers are focusing more on the simultaneous detection of multiple biomarkers for improved disease detection and management. Multiple biomarker detection for diabetes at a time is known as multiplexed detection. It enables the measurement of glucose alongside other diabetes-related biomarkers. Researchers have developed a new paper-based microfluidic sensor that uses α-MnO_2_/graphene quantum dot (GQD) nanocomposites for the purpose of detecting both glucose and L-tyrosine simultaneously. The sensor achieved detection limits of ~0.3 μM for tyrosine and ~58 mg/dL for glucose. Its miniaturized design and integration with portable electronics make it suitable for PoC applications [74].

#### 4.1.3. Cardiac Disease Biomarker Detection

Impedimetric biosensors have emerged as a powerful tool for the rapid and label-free detection of cardiac disease biomarkers. These biosensors are highly beneficial for the early detection and accurate diagnosis of cardiac events such as acute myocardial infarction (AMI). Impedimetric biosensors offer advantages over conventional methods, such as ELISA, which are often expensive, require highly skilled technicians and are not feasible for PoC settings. The key cardiac markers include myeloperoxidase (MPO), myoglobin (Myo), Creatine Kinase-MB (CK-MB) and cTnI, which are critical for the early diagnosis and management of AMI [75,76,77]. Recent advancements depict a wide range of platforms and materials tailored for the detection of cardiac markers. Mondal et al. fabricated a disposable label-free impedimetric biosensor on a polyaniline-coated filter paper substrate for the detection of Myo and MPO. The sensor achieved a LoD of 100 ng/mL in buffer and 500 ng/mL in human serum, with a fast analysis time of less than 20 min. The produced label-free impedimetric biosensor is simple, eco-friendly and low-cost which makes it highly useful for rapid diagnosis, especially in resource-limited settings [75]. In another approach, Kazemi et al. reported a label-free impedimetric immunosensor using porous graphene oxide (PrGO) on a GCE for the detection of cTnI. The anti-cTnI/PGO biosensor was able to successfully detect the cTnI levels in a wide linear range of 0.1 to 10 ng/mL, with an exceptional LoD of 0.07 ng/mL. The outstanding sensitivity of the sensor was achieved due to the high surface area and porosity of PrGO, which facilitates efficient antibody immobilization [76]. Similarly, Tuteja et al. developed a GQD-based impedimetric immunosensor for myoglobin. The sensor achieved a LoD as low as 0.01 ng/mL and demonstrated high specificity even in the presence of potentially interfering and competing proteins. The sensor’s performance was confirmed in spiked human serum samples, with acceptable recoveries between 95 and 105%, indicating its reliability for clinical diagnostics [77].

### 4.2. Impedimetric Biosensors for Environmental Monitoring

Impedimetric biosensors are increasingly recognized as valuable and essential tools for environmental monitoring. They can be used to detect a wide range of environmental contaminants in water, soil, and air.

Impedimetric biosensors are broadly implemented for the detection of heavy metals such as arsenic (As), cadmium (Cd), mercury (Hg), copper (Cu), and lead (Pb) in environmental samples. These sensors use biological recognition elements like aptamers, DNA, enzymes, or whole cells that selectively bind with metal ions, which leads to measurable changes in impedance at the sensor interface. For instance, DNA-based impedimetric sensors form stable metal-DNA complexes. Mercury ion (Hg^2+^) selectively binds thymine (T) bases and creates a thermostable T-Hg^2+^-T duplex, which provides the highly sensitive and selective detection of Hg^2+^ [78]. Another platform for the label-free detection of Lead (II) (Pb^2+^) and Chromium (III) (Cr^3+^) in aqueous solutions biosensor was fabricated by combining a two-dimensional naked-platinum nanoparticles film and DNAzymes, for the detection of Pb^2+^ and Cr^3+^ in the tap water [79]. One of the significant implementations of impedimetric biosensors is in the detection of trace-level pesticides such as atrazine and diazinon. A nanoporous alumina-based impedimetric biosensor achieved ultra-trace detection of atrazine in both drinking and river water with a LoD of 10 fg/mL and a linear range from 10 fg/mL to 1 ng/mL. The sensor possesses high sensitivity because of the nano-confinement of biomolecules within the nanoporous structure, which further amplifies the impedance signal and enhances the efficacy of antigen–antibody interactions [80,81]. Beyond pesticides, impedimetric biosensors can be implemented for the detection of cyanotoxins such as microcystin-LR (MC-LR), a very strong hepatotoxin produced by cyanobacteria. Scientists have produced a 3D-graphene-based impedimetric biosensor in which the functionalization of monoclonal antibodies was performed. The fabricated sensors demonstrated a LoD of 0.05 µg/L for MC-LR-well below the World Health Organization’s guideline for drinking water [82].

### 4.3. Impedimetric Biosensors for Food Safety and Quality Control

A key application of impedimetric biosensors is the detection of foodborne pathogens such as *Salmonella *spp., *Escherichia coli* O157:H7, and *Listeria* monocytogenes. For the detection of foodborne pathogens, impedimetric biosensors often rely on specific biorecognition elements like bacteriophages, antibodies, or aptamers, which are spread over the electrode surfaces. For instance, gold electrodes functionalized with antibodies have provided the means for the detection of *E. coli* O157:H7 at concentrations as low as 3 CFU/mL in complex food matrices like meat and milk. Similar platforms have enabled the detection of *Listeria* monocytogenes down to concentrations of 4–5 CFU/mL [83,84,85]. Recent innovations include the use of bacteriophage-based impedimetric biosensors, which work by exploiting the natural specificity of phages for their bacterial hosts. A recent example is a phage-based nano-biosensor employing a nanocomposite of CNTs, AuNP, and tungsten oxide. The biosensor achieved a LoD of 3 CFU/mL for *E. coli* O157:H7 in various food samples, with rapid response times (as low as 5 min) and high specificity [86]. Moreover, impedimetric biosensors are used in assessing food quality, including monitoring spoilage, freshness and authenticity. Sensors for the detection of spoilage-associated enzymes and metabolic markers enable real-time evaluation of food products such as fish, meat, and dairy, and have proven useful in maintaining quality standards along the supply chain [87].

### 4.4. Impedimetric Biosensors for Agricultural Applications

Agriculture diagnostics, such as pathogen detection, agrochemical residue analysis, and quality assessment, are a primary task for farmers. Thus, Impedimetric biosensors are well adapted for assessing these parameters as they enable real-time monitoring and label-free detection. The portable nature and miniaturized size of impedimetric biosensors also make them a suitable choice for use in agricultural lands. Patel et al. developed an Au-IDE-based impedimetric biosensor for detecting bacterial pathogens in agricultural samples. The biosensor can effectively distinguish between infected and healthy potato tubers by measuring the change in impedance caused by bacterial growth in a nutrient medium. The infected samples demonstrated a discrete impedance profile in comparison to healthy samples, depicting the biosensor’s potential for rapid and field-deployable agricultural disease diagnostics [88]. Likewise, impedimetric immunosensors have been developed for the detection of specific plant pathogens such as *Phytophthora infestans* and *Pseudomonas Syringae*, achieving low detection limits and providing a platform for early intervention [89]. Fungal pathogens such as *Sclerotinia* and *Fusarium* are the cause of rust diseases in plants. Li et al. developed an impedimetric nano-biosensor for the detection of impedance changes in DNA probes for fungal pathogens [90]. Monitoring phytohormone levels and stress responses in plants is essential for optimizing crop management. Impedimetric biosensors can be designed to detect key plant hormones, such as auxin, salicylic acid, and stress biomarkers can be useful for monitoring the abiotic or biotic stress conditions in plants [91].

Table 2 summarizes the application categories reviewed in this section, including citations for the work reported in the literature for each category.

## 5. Potential for Integration with Emerging Technologies

Biosensors, especially impedimetric biosensors, have a great potential for integration with emerging technologies, paving the way for smarter, more responsive, and connected diagnostic platforms. By combining biosensing with advanced computational tools such as AI, ML, the Internet of Things (IoT), and biorobotics, researchers are enhancing data processing, improving analytical accuracy, and enabling real-time, remote health monitoring [92,93,94,95,96,97]. These synergies allow biosensors to transition from standalone devices to components of complex digital health ecosystems. Moreover, miniaturization and advanced materials, as well as state-of-the-art fabrication methods, have emerged, which have allowed these biosensors to be flexible and energy-efficient, thus enabling the development of more effective wearable devices. This convergence supports personalized medicine, predictive diagnostics, and efficient environmental monitoring, making impedimetric biosensors more versatile and impactful in clinical and other applications.

### 5.1. Artificial Intelligence and Machine Learning

The fields of AI and ML have disrupted many fields of science and technology, including the development of novel biosensors, which is opening new avenues for producing more robust and smarter diagnostic and monitoring tools for healthcare, environmental sciences, etc. Although AI applications have recently witnessed exponential growth, very few reports have been published on their integration with biosensing platforms [92].

Recent advances have demonstrated that the use of AI and ML can materially strengthen impedimetric biosensing. This can be achieved by improving signal quality, automating model selection, and extracting parameters. These improvements can enable robust calibration, drift compensation, and direct concentration prediction from complex spectral data [98,99], which addresses some of the key challenges in EIS biosensing as we discussed in Section 2.4. Additionally, these solutions can help in accelerating the translation of lab-scale EIS assays into reliable PoC devices. Some work has already been done on these approaches. For example, Rong et al. used post hoc support vector machines (SVMs) analysis and showed that it can outperform traditional equivalent-circuit modeling for distinguishing weak protein-ligand interactions in impedimetric biosensors, especially when dealing with heterogeneous samples [100]. Similarly, Uzun et al. proposed a framework of stacking regression models enabling robust calibration of biosensor signals while accounting for the environmental and material variability, with high predictive precision as demonstrated through the analysis of 26 ML models for biosensor calibration. Other methods, including principal component analysis (PCA), SHAP, and permutation importance methods, enable ML models to identify the most relevant features from high-dimensional impedance spectra, simplifying biosensor design and reducing costs and analysis times [101]. Furthermore, ML-optimized multiplexing with advanced feature extraction deep learning models has been shown to allow simultaneous detection and classification of multiple biomarkers in a sample [102].

The standard calibration method for impedimetric biosensors typically includes choosing an appropriate equivalent circuit model for the EIS data and applying linear regression between a specific electrode parameter (like ΔR_ct_) and the analyte concentration. ML techniques can streamline this process by automating the selection of the equivalent circuit, thereby minimizing human error. Furthermore, ML algorithms can identify complex, multi-parameter correlations within the high-dimensional EIS data, helping to preserve information that might be lost in single-variable calibration approaches [93,94]. For example, Tayyab et al. developed a fully automated, label-free, ML-assisted platform for protein detection using multi-frequency impedance cytometry integrated within a microfluidic chip. The platform is constructed with cost-effective, off-the-shelf components. The system incorporates a programmable fluid control unit and a custom PDMS-based serpentine microfluidic mixer. The mixer’s performance was validated against a commercial benchtop device using a fluorescent immunoassay. In their work, they targeted salivary IL-6, a key biomarker for oral squamous cell carcinoma (OSCC). Their system detects and quantifies IL-6 via impedance signatures of bead-protein conjugates with 96% accuracy. Another attribute of this platform is that it is adaptable for various proteins by modifying the reagents and bead conjugation protocols [95]. Similarly, Xu et al. leveraged ML tools to develop an ML-enhanced electrochemical impedance EIS biosensor for accurate detection of *E. coli*. Traditional EIS biosensors often rely on tracking changes in single parameters like R_ct_ or C_dl,_ which can be inconsistent across different sensor designs. To overcome this, they immobilized *E. coli* onto gold electrodes via antibody binding and modelled the impedance data using Randel’s circuit to extract multiple electrical parameters. They applied principal component analysis (PCA) and support vector regression (SVR) to correlate these parameters with bacterial concentrations. By integrating multiple features, both capacitive and resistive the ML model significantly improved the accuracy of bacterial quantification compared to conventional single-parameter approaches. This advancement demonstrates the potential of combining EIS with multivariate analysis and ML to develop robust and, automated biosensing platforms suitable for a wide range of pathogen detection and diagnostic applications [93]. Another work reported by Zhang et al. used EIS data along with ML for battery characterization applications. The team collected 20,000 EIS spectra of commercial lithium-ion batteries and used a Gaussian process regression model to predict the effective remaining lifespan and health of the battery [96].

### 5.2. Use in Wearable Devices

Wearable devices have recently gained a lot of traction due to their non-invasive nature, providing real-time diagnostics, data recording and transmission capabilities. Wearable impedimetric biosensors can non-invasively monitor key biomarkers such as glucose, lactate, or stress hormones like cortisol [60,103]. These sensors offer the potential for closed-loop feedback systems that adjust therapeutic interventions in real-time. Their applications far exceed conventional methods of biosensing, enabling features like PoC monitoring, diagnosing, and recording data for further analysis. Additionally, they have the prospect of becoming a key component for AI and ML-enhanced impedimetric biosensors. Their applications are vast and are still being explored for use in healthcare, environmental monitoring, pharmaceuticals, manufacturing, etc.

The work reported in this literature is mostly centered around the detection of biomarkers from the external body fluids, such as sweat [60,97,103]. An example of such work is done by Lee et al., where they developed a conformable, wearable LoP incorporating a stretchable, label-free impedimetric biosensor and microfluidics for on-body cortisol detection in sweat. The device uses 3D-nanostructured gold electrodes integrated into PDMS serpentine microchannels for precise sweat handling, collection, mixing, and waste displacement in a simple “one-touch” operation. During exercise, it accurately quantifies pM-level cortisol concentrations, maintaining quantitative and stable performance. This platform demonstrates the feasibility of flexible, automated systems for continuous biochemical monitoring, and exemplifies the kind of biosensor that could be integrated into micro/nanorobotic systems for autonomous diagnostics [60]. A similar work was reported by Munje et al., where they developed a sweat-based wearable diagnostics biosensor using room temperature ionic liquids (RTILs). One of the major challenges in sweat-based biomarker detection is the reliable targeting of disease-specific small analytes, such as metabolites and proteins. Their detection is complicated by the inherently complex nature of sweat, where variations in pH, ionic strength, and overall composition over time can significantly affect analyte stability and sensor performance. To address these challenges, they used RTILs with antibody-functionalized sensors on nanoporous, flexible polymer membranes. This approach enhanced the stability of antibodies and enabled reliable quantification of proteins. They targeted IL-6 and Cortisol, and employed infrared spectroscopy, dynamic light scattering, and electrochemical impedance spectroscopy to confirm sustained sensor performance. They achieved a LoD for IL-6 of 0.2 pg/mL in the first 24 h, and 2 pg/mL between 24 and 48 h post-functionalization. The sensor also achieved continuous IL-6 detection over 10 h and demonstrated simultaneous detection of IL-6 and cortisol with minimal signal cross-talk [103].

Recent advancements in flexible and nanostructured impedimetric biosensors have significantly expanded the scope of portable diagnostics, enabling real-time, on-body, and highly sensitive detection of pathogenic organisms and protein biomarkers. These systems integrate nanomaterials such as graphene, carbon nanotubes, and silica-based imprints onto flexible substrates, enhancing signal transduction, biocompatibility, and mechanical resilience [104]. In one study, a graphene paper-based immunosensor functionalized with AuNPs and biotin-streptavidin-linked antibodies was developed for the detection of *E. coli* O157:H7. The sensor demonstrated a wide linear detection range (1.5 × 10^2^–1.5 × 10^7^ cfu/mL) and a LoD of 1.5 × 10^2^ cfu/mL, exhibiting robustness against mechanical stress and showing potential for use in low-cost, field-deployable diagnostics [105]. A separate study presented a flexible impedimetric sensor based on gold-coated polyimide for the detection of the inflammatory cytokine TNF-α, relevant to heart failure, and LVAD monitoring. The sensor, which uses antibody immobilization on gold electrodes, operated label-free, achieved a dynamic detection range from 0.1 pg/mL to 0.5 ng/mL, with good selectivity over IL-10 and IL-1, highlighting its utility in cytokine profiling and early inflammation detection [106]. Another flexible platform utilized low-temperature-grown CNTs on polyimide, functionalized with anti-human serum albumin (AHSA) to detect HSA with high specificity. The system used EIS to quantify protein concentrations with a LoD of approximately 30 fg/mL (3 × 10^−11^ mg/mL), demonstrating suitability for wearable and implantable biosensing applications [107]. Additionally, a novel approach utilized molecularly imprinted inorganic silica films for the selective detection of E. coli UTI89. By imprinting bacterial morphology onto gold electrodes, the system achieved ultra-low detection limits (<1 cfu/mL) with a linear range up to 10^4^ cfu/mL. This label-free biosensor provides a promising framework for the development of cost-effective and selective bacterial sensors using artificial biorecognition layers [108].

Flexible and wearable impedimetric biosensing platforms also face some key challenges, such as effective power management, data communication, and biofouling. These platforms must operate at low power to enable continuous, on-body sensing without the need for frequent battery changes or bulky energy sources. Unlike benchtop systems, wearable biosensors typically rely on thin-film batteries, on-body energy harvesting, or rechargeable micro-power modules. As reported by Lee et al., integration of a gold electrode and microfluidics network was employed to produce a stretchable system, which may increase the overall energy demand of the system due to simultaneous sample handling and high-frequency impedance measurement [60]. Reliable data transmission and storage are another challenge, particularly for continuous wearable biosensors. EIS generates complex, multivariate data streams, requiring real-time processing and communication to mobile apps or remote servers. Wireless technologies (Bluetooth, NFC, Wi-Fi) must be power-efficient and robust against interference from the human body or the environment. The limited storage and computational capabilities of flexible devices amplify the need for robust data compression, filtering, and error correction. Advanced machine learning algorithms are increasingly deployed to detect relevant impedance features and reduce data dimensionality before transmission, thus lowering bandwidth and power requirements. Standardized communication protocols and secure data encryption are also essential for maintaining privacy as biosensors migrate into consumer medical devices [109]. Additionally, biofouling is especially problematic in biosensors that utilize sweat or repeated application on skin, where fouling can cause loss of sensitivity and unreliable readings. The antifouling techniques that we discussed earlier can be utilized in wearable devices as well. The reusability of wearable sensors depends critically on the durability of the antifouling layers and the ease of cleaning or replacement [110].

## 6. Conclusions

The field of impedimetric biosensors has rapidly evolved, driven by the demand for sensitive, real-time, and portable diagnostic and biosensing tools in healthcare, environmental surveillance, and food safety domains. This can be attributed to their seamless integration of advanced biorecognition elements with highly engineered electrode architectures, supported by robust and tunable electrochemical techniques like EIS. One of the most striking trends seems to be the shift towards label-free, non-faradaic detection systems, which offer minimal sample preparation time, preserve biomolecule functionality, and enable faster measurements and results. These systems are gaining more attention in applications due to their simplicity and adaptability. The use of nanomaterials such as graphene, MXenes, CNTs, and metal–organic structures has substantially improved sensitivity and lowered detection limits. These materials not only increase electrode surface area but also enhance electron transfer kinetics, offering significant advantages over traditional materials. Similarly, innovative electrode configurations such as IDEs, MEAs, and flexible substrates have enabled miniaturization and integration into wearable and portable systems. In terms of recognition strategies, aptamers and MIPs show promise as robust, scalable alternatives to antibodies and enzymes, especially in harsh environmental conditions. Their stability, ease of synthesis, and reusability make them ideal for long-term monitoring and repeated use in field settings. Additionally, since impedimetric biosensors are founded on a well-established analysis technique, this will help pave the way for receiving regulatory approvals for their future deployment into practical use.

Despite the advancements in this field, several practical and technological barriers still limit the large-scale deployment and commercialization of impedimetric biosensors. The first is stability and reproducibility; many biorecognition elements, particularly antibodies and enzymes, are prone to degradation over time. This leads to inconsistencies and fabrication-related challenges. Advances in micro and nano fabrication methods, as well as electronic fabrication processes, are projected to further advance this class of biosensors by optimizing manufacturing protocols and reducing batch-to-batch variance and quality check requirements.

## Figures and Tables

**Table 1:** Classification, features, and performance parameters of different impedimetric biosensors.

Category /Subtype	Recognition Element	Limit of Detection (LoD)	Time to Response	Anti-Fouling Strategy	Reusability	Sample Matrix	Redox Probe Used	Advantages	Limitations	Reference
Label-free (Faradaic)	Antibody (2,4-D)	0.3 ppb	Not reported	PANABA/MWCNT/AuNP	No	Water, agro Samples	Yes	Selectivity, sensitivity, robust signal	Needs a redox probe, single use	[55]
Label-free (Non-faradaic)	DNA probe (Avian Influenza)	1 fM	Not reported	Dielectric film, APTES/Glutaraldehyde	No	Buffer, serum	No	Real-time, reagent-free	Lower sensitivity	[56]
Label-free (Faradaic)	Aptamer (Salmonella)	6 CFU/mL	30 min	Diazonium-polymer	No	Apple juice, buffer	Yes	Real sample validated, ultra-low LoD	Needs a redox probe, single use	[5]
Label-free (Faradaic)	Aptamer (DXN)	2.12 nM	Not reported	MCH	No	Milk, buffer	No	Reproducible, selective, food compatible	No active antifouling	[57]
Label-free (Non-faradaic)	MIP (Exosome, NSCLC)	2.03 × 10^3^ particles/mL	Not reported	MIP layer	Yes	Cell, serum	No	Selectivity, reuse, stability	Complex synthesis	[6]
Label-free (Non-faradaic)	Antibody (IL-8)	90 pg/mL	Not reported	Au-IDE, Z_imag_ analysis	No	Serum, buffer	No	Sensitive cytokine detection	Single use	[30]
Label-free (Non-faradaic)	Antibody (LDL cholesterol)	120 pg/mL	Not reported	Not reported	No	PBS, blood	No	Wearable integration, continuous	Low stability and specificity	[59]
Label-free (Non-faradaic)	Spike protein (SARS-CoV-2)	0.4 BAU/mL	<1 h	Ionic strength tuning	No	Serum	No	PoC/serology, label-free	Ionic dependence	[34]
Label-free (Non-faradaic)	Nucleocapsid (SARS-CoV-2)	0.1 ng/mL	60 min	ToAD 96-IDE array	No	Serum	No	High throughput, multiplexed	Multiplexing complexity	[54]
Label-free (Non-faradaic)	Cortisol antibody	pM range	Not reported	Stretchable microfluidic, AuNS	Yes	Sweat	No	Reusable, non-invasive, robust	Fabrication cost	[60]
Label-free (Non-faradaic)	VEGF antibody	1.03 pg/mL	Not reported	Biodegradable silk polymer	Yes	Buffer serum, simulated urine	No	Biocompatibility, eco-friendly	single use	[53]
Label-free (Non-faradaic)	Chemotactic capture (zoospore)	Not reported	Not reported	Microfluidics	Yes	Plant water, field sample	No	Field detection, high selectivity	Low throughput	[62]
Label-based (Faradaic)	Lectin (CEA/Bacteria)	0.01–0.05 ng/mL	Not reported	Lectin array multiplexed MEA	No	Blood	Yes	Multiplexing, robust signal	Probe/addition needed	[63]
Label-based (Faradaic)	Antibody-AuNP (E. coli)	100 cfu/mL	Not reported	AuNP-modified SAM	No	Buffer, water	Yes	Signal-off, nanoparticle amplification	Nanoparticle requirement	[66]
Label-based (Faradaic)	GOx enzyme (Glucose)	27 μM	<10 s	CuO-chitosan nanocomposite	No	Serum	Yes	Fast, enzyme-amplified, robust	Enzyme stability	[68]
Label-based (Faradaic)	Magnetic nanobead/ALP (Salmonella)	50 cfu/mL	3 min	Magnetic separation, microfluidic	No	Spiked chicken supernatant	Yes	Fast, sensitive, multiplexing	Bead-label integration complexity	[67]

**Table 2:** Applications of impedimetric biosensors.

Domain	Target(s)	Recognition Element/Platform	Format	References
Medical/Clinical	PSA, cTnI, IL-6, VEGF, Myoglobin, MPO, CK-MB	Antibodies, aptamers, nanocomposites	Label-free (mostly Faradaic)	[75,76,77]
	Glucose, Tyrosine	Enzymes (GOx), nanocomposites	Label-based Faradaic	[68,69]
	Exosomes	MIP-based GCE	Label-free Faradaic	[6]
Environmental Monitoring	Herbicides (2,4-D), heavy metals, pesticides, toxins	Antibodies, aptamers, nanostructures	Both	[78,79,80,81,82]
Food Safety	*Salmonella*, *E. coli*, and antibiotic residues	Aptamers, antibodies, phages	Both	[83,84,85,86,87]
Agriculture	Airborne ascospores, plant pathogens	IDEs, DNA probes	Label-free non-faradaic	[88,89,90,91]
Wearables/Flexible	Cortisol, VEGF, cytokines	Stretchable Au electrodes, silk-sericin inks	Label-free non-faradaic	[53,60]

## Data Availability

No new data were created or analyzed in this study.
